# The revitalization of “Osekkai”: How the COVID-19 pandemic has emphasized the importance of Japanese voluntary social work

**DOI:** 10.1177/1473325020973343

**Published:** 2021-03

**Authors:** Ryuichi Ohta, Akiko Yata

**Affiliations:** Community Care, 73828 Unnan City Hospital, Unnan, Japan; Community Nurse Company, Unnan, Japan

**Keywords:** COVID-19, aging, social work practice, rural social work, Japan, Osekkai

## Abstract

Coronavirus disease 2019 (COVID-19) has caused worldwide panic, and rural areas are no exception. In Japanese rural areas, many older people live alone and lack access to reliable sources of information. During the pandemic, older adults were initially isolated from their communities because of the recommended social isolation measures, even when there were no cases in rural communities. However, various formal and informal caregivers went beyond their usual roles and tried to reconnect the older rural population with their communities and nurtured their social connections; Japanese community workers mitigated the stress and fear experienced by the rural elderly in the COVID-19 pandemic. Furthermore, this pandemic encouraged rural Japanese customs. One such custom is “Osekkai.” The Japanese word Osekkai describes actions that someone considers useful and meaningful to perform for others. Osekkai involves both formal and informal care, and as social gatherings began to disappear, Osekkai allowed individuals to deal with the various social problems created by the pandemic. Conferences based on Osekkai can strengthen rural people’s connections and improve their social capital. Activities of rural people that are constructed through Osekkai conferences are not only evidence-based but also based on reliance. This unprecedented pandemic has taught us not only the importance of usual healthcare and precautions against infection but also that nurturing social connection in communities is crucial in the face of social turbulence.

## Introduction

The coronavirus disease (COVID-19), caused by the novel coronavirus, has become prevalent worldwide, negatively affecting human beings both physically and mentally. COVID-19 first appeared in China in December 2019, and within a few weeks, a pandemic was declared by the World Health Organization (Yang et al., 2020). Travelers and workers rapidly transmitted the virus from China to the rest of the world (Yang et al., 2020). Although most countries have tried to stop the spread of COVID-19 with radical methods, such as quarantines and national lockdowns, there have been hundreds of thousands of fatalities resulting from the acute respiratory syndrome caused by COVID-19 (Remuzzi and Remuzzi, 2020). This infection has also affected the mental health of people all over the world. Regardless of profession, people, especially healthcare professionals, might experience depression resulting from the various restrictions imposed and the constant pressure of potentially contracting the disease (Wang et al., 2020).

The approaches to combat COVID-19 have varied according to each country’s culture and governance. In Japan, although there were many infected patients on a Princess Cruises’ ship in Yokohama called the Diamond Princess in late February 2020, the epidemic did not spread to other regions (Mizumoto et al., 2020). As the spread of the virus through Japan was expected to be relatively delayed due to inherent cultural social distancing and collectivism (Hofstede, 2011), a lockdown of the country was not carried out. However, there was a gradual increase in the number of COVID-19 patients, mainly in urban areas such as Tokyo and Osaka.

The fear of COVID-19 spread all over Japan. In rural areas, although there were few cases, people were stressed by the invisible virus. The situations in rural areas are completely different from urban areas. The built environment and population density in Japanese rural areas are lower than in urban areas; therefore, there are fewer opportunities for physical contact with others. However, rural residents were forced to behave in the same way as urban residents. Many rural Japanese, including community social workers and healthcare professionals, were confused with the news from TV and social media. They struggled with managing their fears and tried to collaborate to overcome this unprecedented pandemic by utilizing various human resources in their rural communities. This essay first describes the Japanese rural population’s social distancing in the pandemic, highlighting their experiences of stress and fear. Next, the problems of COVID-19 mitigation approaches in rural areas are described, along with some original solutions by indigenous formal and informal workers. This essay describes our community’s social activity in the pandemic of COVID-19. This social activity can provide a novel method of social work to support and motivate rural people during the COVID-19 pandemic. Considering that the world is aging and there are various rural communities struggling with the pandemic, this realistic approach can be applied there.

## Natural social distancing and bridging informal support in rural areas

Japanese rural populations are shrinking gradually. The average age in rural areas is also increasing because of the low birth rate and migration of young people to urban areas (Muramatsu and Akiyama, 2011). The older people who remain in rural areas have decreased access to social opportunities because of their low mobility (Tanaka and Iwasawa, 2010). Older people, especially those over 75 years old, are encouraged to relinquish their driving licenses because of the increase in the number of car accidents involving older people in urban areas (Ichikawa et al., 2016). As this trend affects lives outside of urban areas, more and more rural elderly are relinquishing their drivers’ licenses, increasing their isolation from society and decreasing their access to social opportunities. In addition, access to information is also restricted by the limited availability of information sources in rural areas (Ohta et al., 2020). Older people often depend only on newspapers, televisions, and calls from their family members for information regarding healthcare and infection. A lack of access to information can cause delayed reactions to infection and inappropriate behaviors based on fake news. The rural elderly who live alone may find it more difficult to deal with their health and approaches to mitigate infection.

Formal and informal community support forms the foundation of interpersonal relationships among older people in rural areas, which was virtually destroyed by the COVID-19 pandemic. In the usual care of older rural populations, Japanese public health nurses visited older peoples’ homes, shared each community’s conditions, such as indigenous events and various epidemics, and monitored their health conditions, especially noting acute or chronic symptoms impinging on their lives (Yamashita et al., 2005). These activities kept older people up to date with knowledge regarding their own health care and local epidemics. Compared to urban areas, Japanese rural people suffer from a knowledge gap regarding healthcare issues from healthcare professionals (Ohta et al., 2020). Informal care from other rural people who form community organizations to visit older peoples’ homes helped to fill that gap, and space was provided for older people to exercise and socialize. These measures sustained their health and sense of a life worth living (Ohta et al., 2019).

However, the COVID-19 pandemic has changed the rural elderly’s lives. The Japanese government suggested that all citizens sequester themselves to prevent spreading the virus. Fewer and fewer people left their homes, and social interactions among people drastically decreased in shopping malls and restaurants. In rural areas, families would commonly visit their parents, but many young people restrained themselves by considering the possibility of spreading the infection. The older rural population tended to experience more fear of the virus because of its high mortality rate in older people. Various formal and informal care measures have decreased because of the fear of potential spread, even when caregivers have not had any exposure to infected patients. All these factors have contributed in deteriorating the rural elderly’s lives and isolated them even more than before the pandemic.

## Inappropriate stress from COVID-19

**Figure 1.**
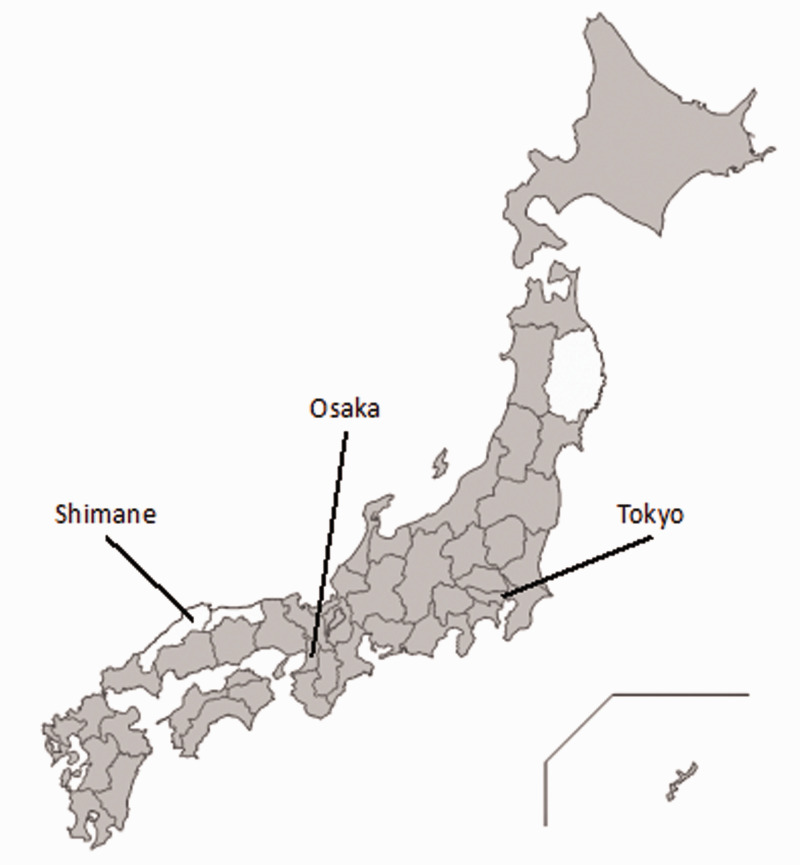
Spread of COVID-19 in Japan as of April 7, 2020. Grey-colored prefectures had at least one COVID-19 patient.

The stress from COVID-19 was so tremendous that rural communities panicked even though there were no patients in our prefecture, Shimane (Figure 1). Clinical pandemic information and stories of personal experiences with COVID-19 in Japanese urban areas such as Tokyo and Osaka circulated throughout Japan, overwhelming rural people. Depending only on the news from TV and newspapers, which presented sensational headlines, the older rural population experienced information overload and lack of appropriate information (Ohta et al., 2020).

In addition, the application of the urban framework of social distancing approaches, such as the suppression of meeting neighbors and families, in rural areas may have decreased rural peoples’ mental health. Although standard preventions for COVID-19 were prescient, such as handwashing and wearing a face mask (Feng et al., 2020), extreme social distancing made the rural elderly panic and experience inappropriate stress. Those in rural areas might have felt cut off from society, feeling isolated and growing depressed because the pandemic seemed endless as the number of patients gradually increased. Everybody was hoping for ways to collaborate and ease the stress and fear in rural communities.

## The necessity of voluntary collaboration among rural people through “Osekkai”

Japanese social workers turned to traditional practices to mitigate the stress and fear of older rural populations during the COVID-19 pandemic. One traditional Japanese custom is “Osekkai,” a Japanese word that describes “actions which someone considers useful and meaningful to do for others.” Osekkai can sometimes be regarded as intrusive and redundant when it is applied to sensitive situations or when people do not need any help. However, emergent situations require individuals’ voluntary activities for communities in which human resources can be limited and isolated people are not able to seek help. Various forms of Osekkai are performed in rural areas. Some people voluntarily go around their towns and confirm whether people are healthy or facing difficulties by asking them. Others may take older people to medical institutions or call the medical institutions on their behalf because they understand that the older people may be unhealthy even when they do not seek help on their own.

In this pandemic, many rural elderly, especially those living alone, could not receive reliable information or express the difficulties, fear, or growing stress they were experiencing from COVID-19 because of the lack of internet access. Various forms of Osekkai, including informal and formal caregiving, were used to connect with the rural elderly in their communities. Different formal and informal care arrangements gradually emerged from the dark situation. Caregivers reached out to rural elderly, and they went beyond their usual roles to help in the pandemic. Rural medical physicians visited rural communities to provide appropriate information about the pandemic and the condition of the communities. Post office workers checked on the health conditions of the older rural population and conveyed that information to community health and social workers. Community nurses and health workers voluntarily visited rural people’s houses or brought them to community centers to provide reliable information on the recommended methods for preventing the spread of COVID-19 and encourage them to persevere in their difficult situations. In addition, both formal and informal rural care providers established flexible meetings called the Osekkai conferences, which allow people to get together and discuss community problems by sharing knowledge. The conferences take place in small groups to avoid spreading COVID-19 in rural communities.

**Figure 2.**
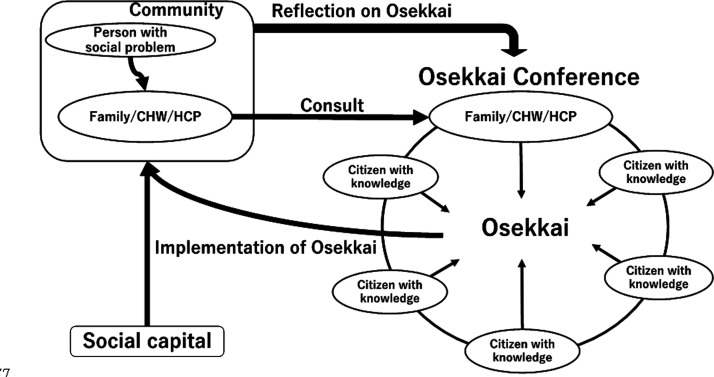
The conceptual framework of the Osekkai conference. CHW: community health worker; HCP: healthcare professional.

At the Osekkai conferences, various rural people present their community’s problems and social issues. The conferences involve multiple people with different professional backgrounds, such as medicine, law, public health, rehabilitation, architecture, community development, and transportation. Regarding community problems, conference attendees work collaboratively to solve problems by sharing their own experiences and suggesting solutions to similar problems. Finally, an Osekkai plan is established. The plan is then carried out in each rural community with the suggested resources. The results of the plan’s provision are then shared in the following Osekkai conferences. Through continual discussions, Osekkai plans can be revised, and the quality of care can be improved. Furthermore, the constant discussions can foster new, effective collaborations between providers of different resources (Figure 2).

## Examples from Osekkai conferences

An Osekkai conference enabled one older woman with a disability from a neurodegenerative disease to be involved in her community and increase her social capital during the COVID-19 pandemic. This older woman was unable to go out and buy her groceries by herself. She was originally a manager of a food shop and made food for people in rural communities. She loved her job but had to resign because of her disease. She used long-term insurance-based care, which was supported by the Japanese social insurance system. Every week, home care nurses visited her to manage her symptoms. She expressed the desire to buy her groceries by herself to her home care nurses, as buying groceries was one of her previously essential routines. In the Japanese system, home care nurses cannot go with patients to other places. Because of the COVID-19 pandemic, home care nurses and home care workers could not visit her frequently. After hearing her desire, one of the home care nurses brought the issue to the Osekkai conference. At the conference, attendees discussed the problems associated with the mobility issues caused by her disease. One of the participants suggested the use of a new delivery service by a person with a nursing license as a resource. Another participant shared that one shopping mall had rehabilitation programs based on using bodily motion while shopping. Through the discussion, an Osekkai plan was established for the woman and prepared for implementation without relying on formal care.

The provision of the plan presented another challenge because of a lack of resources. The other day, the plan was performed, and the result was successful. The older woman was satisfied and hoped to use this plan continuously. However, the resources of the rehabilitation and delivery services were limited, so the plan could not be performed continuously. The following conferences focused on the woman’s social connections that she had developed from her previous work. She had various relationships with people of many different backgrounds in her community. Among her connections, several people were willing to join the plan and take her to the mall. Nursing staff taught these volunteers how to move her. Eventually, she was able to continue to get her own food by getting help from a few community members providing informal care. In this situation, formal care, which was unable to function well during the pandemic, could pass social problems to informal care effectively. Furthermore, this Osekkai not only empowered the woman but also bonded and bridged various human resources around her, which may improve the social capital within the community.

## Reflection in the pandemic

### The importance of Osekkai

Collectivism, a traditional characteristic in Asian regions and island countries related to Buddhism, can drive activities of Osekkai (Hofstede, 2011). In collectivist societies, people respect each other’s ideas, refrain from insisting on their own opinions, and can effectively collaborate as a group (Hofstede, 2011). There are various arguments against collectivism because it is less respectful of original ideas and engenders feelings of suppression caused by the enforcement of traditions (Yamagishi et al., 1998). However, reflecting on this pandemic in Japan, mutual understanding and collaboration through face-to-face communication based on Osekkai might have the capacity to revitalize rural communities and empower vulnerable rural people. In rural societies, although there is a lack of resources, social workers can voluntarily collaborate and overcome this difficulty, which shows the importance of collectivism in emergencies.

### The effect of the Osekkai conference

The Osekkai conference as social movement has the possibility to drive rural community sustainability through more mutual assistance. This conference has two objectives: solving social problems in a community and nurturing relationships between community members. The Osekkai conferences provide people in rural communities with the platforms needed to openly discuss their social problems. Conference participants are people familiar with the local resources, backgrounds, and cultures of their own communities. The participants are able to freely discuss local issues based on their common ground. As such, their discussions foster possible solutions in achievable forms.

Osekkai conferences can bind and reorganize social capital in rural communities. Through these conferences, different resources are connected through discussion of the social issues in each community. Although an overall lack of resources remains, the multiple connections of existing resources can provide realistic solutions for communities, supported by professional opinions (Ohta et al., 2020). The connection of resources can lead to social capital, which reflects resources that people can access in effectively managing their issues. These conferences can not only solidify personal social capital but also build bridges among people who did not previously have any connections (Eriksson, 2011).

### For the preparation of coming social turbulence

Emergent situations bring to light the importance of voluntary social work, which involves various resources based on experience and evidence. In a pandemic, people have to follow evidence-based approaches, such as wearing a face mask, washing hands, and practicing social distancing. While people should do their best to not spread the disease, they should continue to rely on each other to nurture and sustain the social connections and support in their communities. In Japanese rural areas with high natural social distancing, people who practice evidence-based prevention techniques can collaborate to support their communities, regardless of whether or not they are healthcare professionals.

During the current pandemic, credit to rational community health workers and other professionals, panic in Japanese rural communities has been avoided, and older people in rural areas were provided with appropriate information about COVID-19. This unprecedented pandemic has taught us not only about the importance of customary healthcare practices and precautions against infection but also of nurturing the social connections in communities, which can be flexible and effective in the face of social turbulence, using the example of Japanese traditional social work Osekkai.
